# Regeneration of invariant natural killer T (iNKT) cells: application of iPSC technology for iNKT cell-targeted tumor immunotherapy

**DOI:** 10.1186/s41232-023-00275-5

**Published:** 2023-05-12

**Authors:** Takahiro Aoki, Shinichiro Motohashi, Haruhiko Koseki

**Affiliations:** 1 Laboratory for Developmental Genetics, RIKEN Center for Integrative Medical Sciences, Yokohama, Japan; 2 Department of Medical Immunology, Graduate School of Medicine, Chiba University, 1-8-1 Inohana, Chuo-Ku, Chiba, 260-8670 Japan; 3 Department of Cellular and Molecular Medicine, Graduate School of Medicine, Chiba University, Chiba, Japan

**Keywords:** iNKT cells, iPS cells (iPSCs), Cancer immunotherapy, Innate immunity, Acquired immunity, Clinical trial

## Abstract

Invariant natural killer T (iNKT) cells are a subset of innate-like T cells restricted by a major histocompatibility complex (MHC) class I-like molecule, CD1d. iNKT cells express an invariant T cell receptor (TCR) encoded by Vα14 Jα18 in mice and Vα24 Jα18 in humans and are activated by recognizing glycolipid antigens, such as α-galactosylceramide (αGalCer), presented by CD1d. iNKT cells exhibit anti-tumor activity via their NK-like cytotoxicity and adjuvant activity. Although iNKT cell-targeted immunotherapy is a conceptually promising approach, we still found a technical hurdle for its clinical implementation which is mainly due to the low frequency of iNKT cells, particularly in humans. To compensate for this, we proposed to generate adequate numbers of clinically competent NKT cells from induced pluripotent stem cells (iPSCs) for cancer immunotherapy. Toward this goal, we first obtained the proof of concept (POC) for this approach in mice. We developed a technology to differentiate iPSCs into iNKT cells (iPSC-iNKT cells) and found iPSC-iNKT cells efficiently rejected a syngeneic experimental thymoma by inducing antigen-specific CD8 T cells. After achieving the POC in mice, we developed human iPSC-iNKT cells, which had a high correlation in their gene expression profiles with parental iNKT cells. Human iPSC-iNKT cells also exhibited anti-tumor activity and adjuvant activity for human NK cells in vivo. Based on this supporting evidence for the anti-tumor activity of human iPSC-iNKT cells, we began to generate good manufacturing practice (GMP)-grade iPSC-iNKT cells. As of now, the first-in-human clinical trial of iPSC-iNKT cell therapy is ongoing as a single-agent, dose-escalation study for patients with advanced head and neck cancer. Demonstration of the safety of iPSC-iNKT cell therapy may allow us to improve the strategy by further reinforcing the therapeutic activity of iPSC-iNKT, cells either by gene-editing or combinatorial use with other immune cell products such as dendritic cells. Sixteen years after the establishment of the iPSC technology, we are reaching the first checkpoint to evaluate the clinical efficacy of iPSC-derived immune cells.

## Background

Invariant natural killer T (iNKT) cells are a subset of innate-like T cells sharing some characteristics with NK cells and restricted by a major histocompatibility complex (MHC) class I-like molecule, CD1d. Most NKT cells express an invariant T cell receptor (TCR) encoded by Vα14 Jα18 in mice [1] and Vα24 Jα18 in humans [2, 3], paired with a limited number of TCRβ chains Vβ8.2, 7, and 2 in mice and Vβ11 in humans; thus, the name invariant NKT (iNKT) cells. Unlike conventional αβT cells, which recognize peptide antigens presented by MHC class I/II complexes, the iNKT TCR recognizes glycolipid antigens presented by CD1d [4]. Later, a glycolipid, α-galactosylceramide (αGalCer), was identified as an activating ligand for iNKT cells in mice and humans [5]. Importantly, although CD1d is an MHC class I-related molecule, it is monomorphic in each species, in sharp contrast to the highly polymorphic MHC class I molecules [6]. This implies that αGalCer can uniformly activate iNKT cells irrespective of the vast diversity of MHC class I and II in both humans and mice.

Activities of iNKT cells have been repeatedly addressed by using mouse models in which iNKT cells are depleted by deleting the *J*α*18* gene segment or *Cd1d* [7, 8]. In *J*α*18*-knockout (KO) mice, we observed that IL-12- and αGalCer-mediated tumor rejection was fully disrupted [9]. IL-12 is well accepted to be a critical regulator for host defense by coordinating innate and adaptive immunity. iNKT cells were found to be essential mediators for IL-12 signaling to optimize host defense mechanisms. Similarly, tumor rejection by αGalCer-pulsed dendritic cells (hereafter αGalCer/DCs) was disrupted in iNKT cell-deficient mice, again suggesting the anti-tumor activity of iNKT cells. It is also noteworthy that the incidence of methylcholanthrene-induced tumor development was higher in iNKT cell-deficient mice [10]. This finding indicates that iNKT cells are active under physiological conditions, in which overt exogenous stimuli to activate iNKT cells are lacking. In accordance with the anti-tumor activity of iNKT cells observed in the mouse model, the failure of iNKT cell reconstitution after stem-cell transplantation for leukemia was reported to be associated with relapse [11]. Importantly, iNKT cells are also reported to play an important role in host defense against a variety of microbial pathogens. For example, pulmonary challenge with *Pseudomonas aeruginosa* in *Cd1d*-KO mice resulted in bacterial overgrowth at the early stages of infection [12]. These and many other observations indicate a pivotal role of iNKT cells for host defense and prompted us to consider iNKT cells as a plausible target for anti-cancer immunotherapy. In this review, we will summarize our longstanding challenges towards clinical implementation of the anti-tumor therapeutic potential of iNKT cells via the benefit of iPSC technology.

## iNKT cell modes of action to eliminate tumor cells: activation of NK cells and antigen-specific T cells

**Fig. 1:**
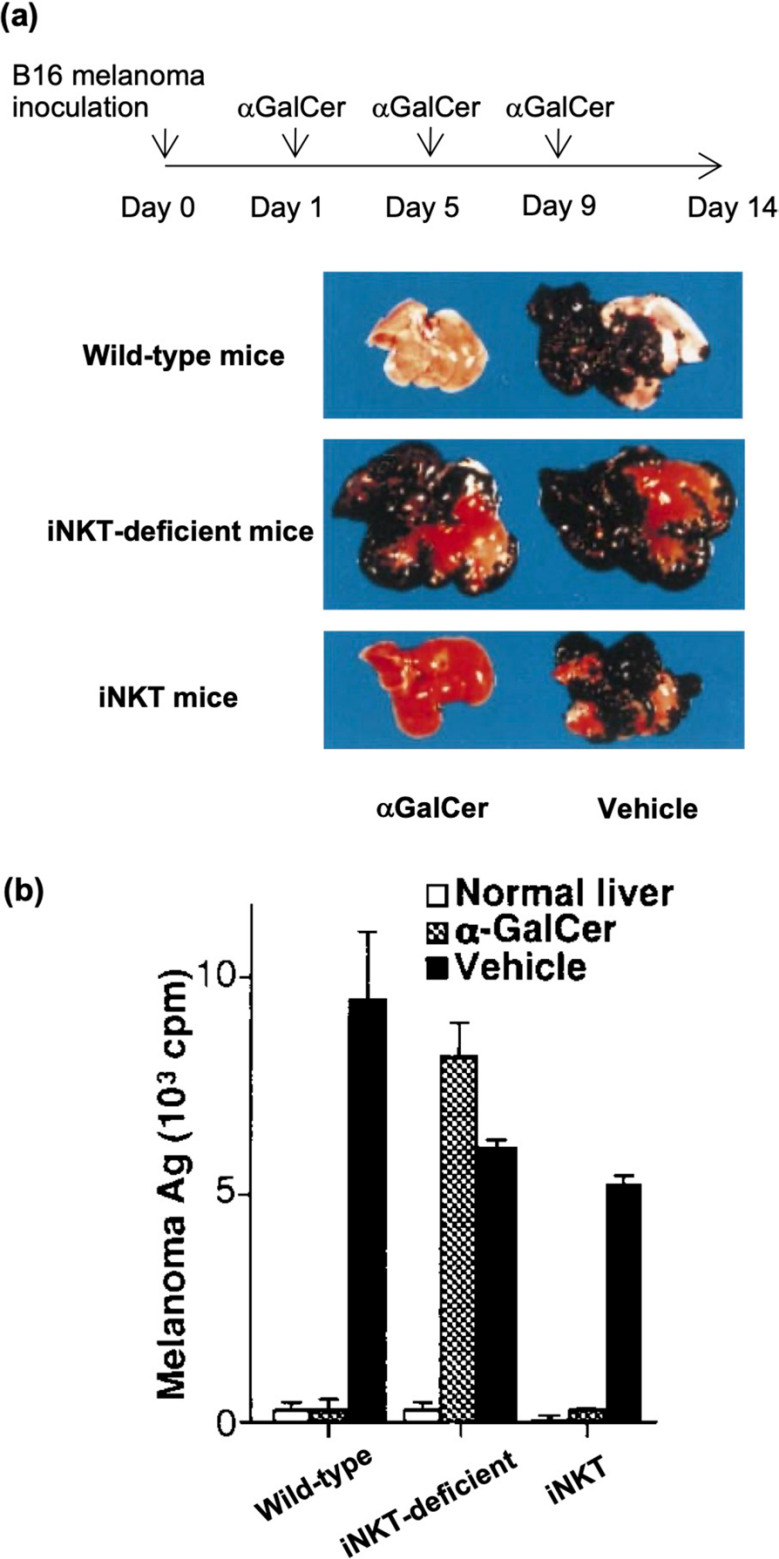
In vivo anti-tumor effect of iNKT cell-targeted immunotherapy. **a** Metastasis of B16 melanoma after administrating αGalCer or vehicle in wild-type, iNKT-deficient (*J*α*18*-/-), and iNKT (*Rag2*-/-, *V*α*14/Vβ8.2* TCR transgenic) mice. **b** Melanoma antigens in metastasized livers measured by radioimmunoassay. Figures are adapted with permission from Kawano et al., PNAS, 1998 [13], Copyright (1998) National Academy of Sciences, U.S.A. (PNAS is not responsible for the accuracy of this translation.)

The therapeutic potential of iNKT cells for cancer is supported by their multiple action modes to activate anti-tumor immunity. Our previous study using the B16 melanoma cell line liver metastasis model revealed that ligand-activated iNKT cells exerted direct cytotoxicity to B16 cells [13]. Importantly, B16 cells were efficiently eliminated in mice lacking T, B, and NK cells but possessing iNKT cells (*Rag2*-KO with *Vα14*/*Vβ8.2* TCR transgenes). Conversely, αGalCer-induced elimination of B16 cells was disrupted in iNKT-deficient mice (Jα*18*-KO mice) (Fig. 1). These observations indicate that activated iNKT cells can efficiently eliminate B16 tumor cells on their own upon αGalCer-mediated activation. Although iNKT cell-mediated direct cytotoxicity is expected to involve perforin, since it was abolished by the perforin cytotoxicity inhibitor concanamycin A [13], its precise mechanism is still controversial. Cytotoxicity of iNKT cells does not require CD1d expression on cancer cells, which means that iNKT cells do not use their invariant TCR to exert cytotoxicity for target tumor cells. However, this seemingly NK-like cytotoxic activity was not associated with Ly49C/NK1.1 molecules either, even though these are essential for conventional NK cell-mediated cytotoxicity. Consistently, unlike NK cells, iNKT cells can eliminate tumor cells expressing MHC class I [13]. Therefore, iNKT cells may recognize tumor cells using a different mechanism from T cells or NK cells to exert anti-tumor effector activity.

However, such cytotoxic activity for tumor cells is not necessarily considered to be a major part of the anti-tumor activity of activated iNKT cells. Instead, iNKT cells contribute to tumor cell elimination by activating many other immune cell types upon stimulation by αGalCer/DCs. Activated iNKT cells immediately produce abundant interferon-γ (IFNγ), which primarily activates NK cells to eliminate MHC-negative tumor target cells [14, 15]. This NK cell activation is followed by the induction of antigen-specific CD8 and CD4 T cells via their clonal expansion. These expanded T cells are expected to facilitate the elimination of tumor cells expressing canonical MHC molecules and also the induction of anti-tumor immunological memory. In parallel with the activation of NK cells and T cells, iNKT cells facilitate DC maturation [16]. DCs matured by activated iNKT cells are expected to reciprocally enhance protective innate and acquired immune responses. Taken together, upon activation by αGalCer/DCs, iNKT cells can activate multiple anti-tumor immune pathways, which likely synergize with each other. Therefore, iNKT cells are a critical cellular component of the anti-tumor adjuvant activity.

## iNKT cell-targeted immunotherapy in advanced cancer patients

The abovementioned anti-tumor adjuvant activity of activated iNKT cells encouraged us and others to examine the anti-tumor therapeutic potential of iNKT cells in humans [17–20]. We indeed launched clinical trials of iNKT cell-targeted immunotherapy for advanced lung cancer and head and neck cancer at Chiba University Hospital [21]. In our phase I clinical trial for non-small cell lung carcinoma (NSCLC), patients were intravenously challenged with autologous αGalCer-pulsed antigen-presenting cells (αGalCer/APCs) including DCs [22]. In this phase I study, safety profiles of αGalCer/APCs were examined at three different doses: level 1: 5 × 10^7^, level 2: 2.5 × 10^8^, and level 3: 1 × 10^9^ cells/m^2^. Patients who received the level 3 dose of αGalCer/APCs exhibited increased iNKT cells in the peripheral blood that were accompanied by an increase in the median survival time (MST). This result was recently reproduced in a phase II study [23]. A total of 35 patients were enrolled, and the MST was 21.9 months (95% confidence interval, 14.8–26.0). This result was promising in the cohort of pretreated NSCLC patients who received first-line chemotherapy because the MST of recent second-line therapy with immune checkpoint inhibitors was 9.2–12.6 months [24–26]. The immunological monitoring in this study revealed a significant increase in the number of NK cells and effector CD8 T cells. In addition to our studies, several clinical trials were conducted at other medical institutions [27]. Richter et al. reported on a phase I study of αGalCer-loaded monocyte-derived DCs in combination with the immunomodulatory drug, lenalidomide performed against myeloma [28]. Broad immune activation, such as iNKT cell reduction and NK cell and monocyte activation, was detected after the combination therapy. A reduction in the monoclonal immunoglobulin produced by the tumor cells was found in 3 out of 4 patients.

Immunotherapy using autologous αGalCer/APCs was further extended to head and neck cancer (HNC) via nasal submucosal injection [29]. In this case, the clinical efficacy of the treatment turned out to be limited. However, complementation with autologous iNKT cells, which had been expanded in vitro, was shown to compensate for the limited effects of αGalCer/APCs [30]. In this phase I study, some of the patients challenged by two nasal submucosa injections of αGalCer/APCs (1 × 10^8^ cells/injection) and one intra-arterial infusion of ex vivo expanded iNKT cells (5 × 10^7^ cells/injection) exhibited a partial response (PR). In the phase II study [31], ten patients with locally recurrent HNC were treated in the same way and some clinical efficacy was observed in all patients; PR in five patients and stable disease (SD) in the other five. The frequency of iNKT cells among tumor-infiltrating mononuclear cells correlated with the clinical outcomes to some extent; it was higher in PR cases than in SD cases, supporting a therapeutic impact of exogenously complemented iNKT cells. These results indicate that activation of either endogenous and/or exogenous iNKT cells by αGalCer/APCs exerts anti-tumor activity in humans.

## Complementation of iNKT cells derived from iPSCs: proof of concept in mice

The clinical trial for HNCs revealed that exogenous iNKT cells can be activated by αGalCer/APCs and enhance the anti-tumor therapeutic efficacy in humans [31]. This prompted us to explore whether iNKT cells could be more efficiently produced on an industrial scale. We then came upon the idea that iPSCs could be a good source of functionally competent iNKT cells. This possibility was first tested in mice and then extended to humans.

**Fig. 2:**
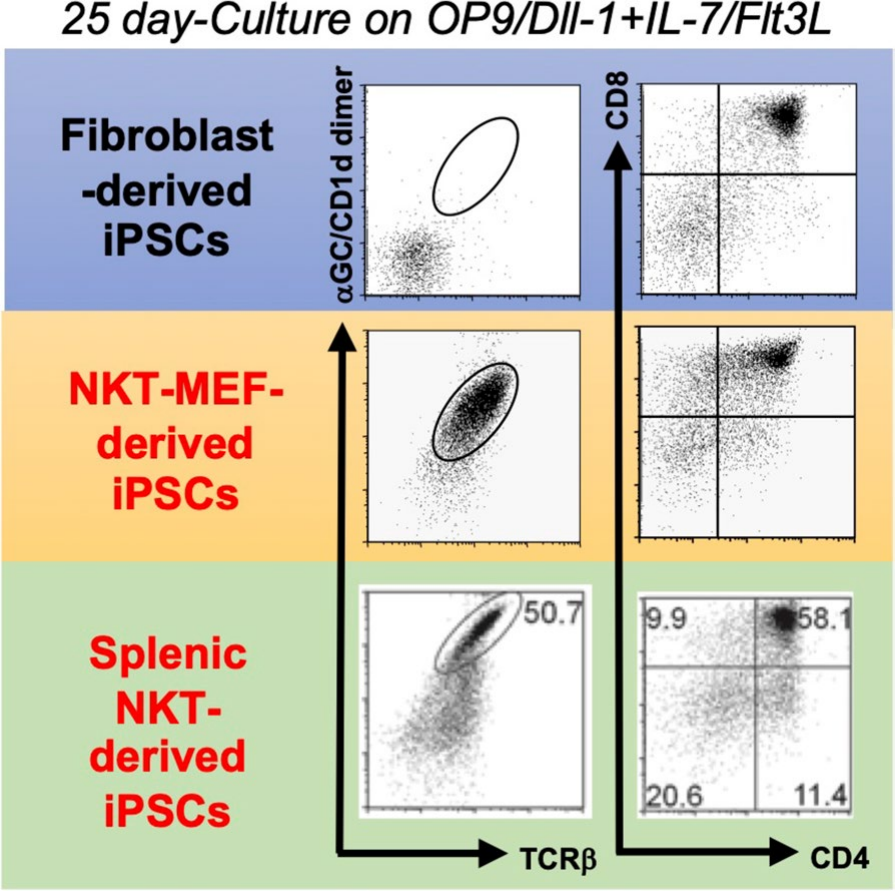
Development of iPSC-iNKT cells in mice. Differentiation of iPSCs of different origins into iNKT cells. Figures are adapted with permission from Watarai et al., J Clin Invest, 2010 [35], Copyright (2010) American Society for Clinical Investigation

**Fig. 3:**
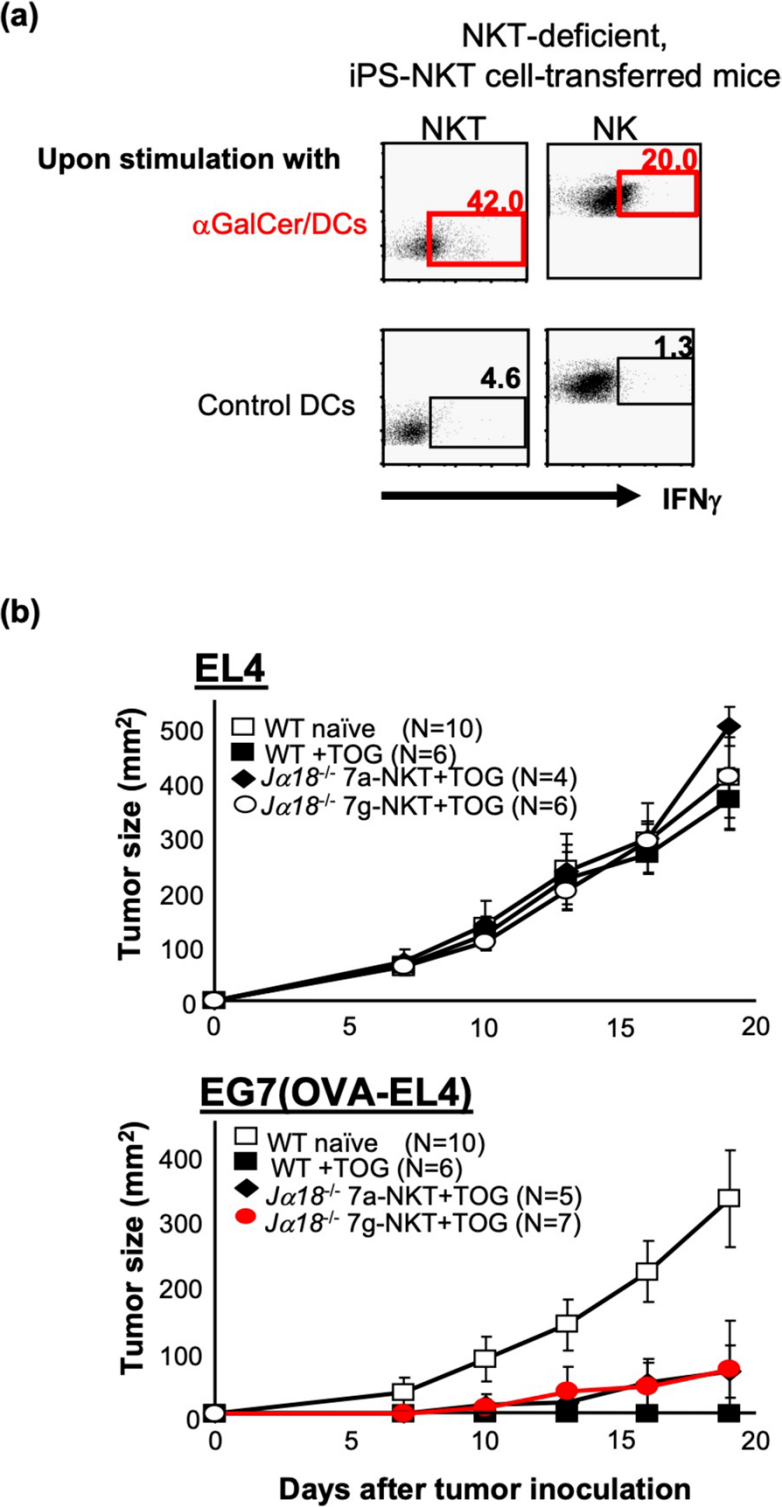
In vivo adjuvant activity of iPSC-iNKT cells in mice. **a** Adjuvant activity on NK cells induced by iPSC-iNKT cells upon stimulation using αGalCer/DCs. **b** In vivo tumor cytotoxicity via the adjuvant effect of iPSC-iNKT cells in an ovalbumin (OVA)-dependent manner. EL4 is a B6-derived thymoma line and EG7 is the OVA-expressing EL4 subline. TOG is the administration of OVA-loaded dying spleen cells from TAP^–/–^ mice with αGalCer. Figures are adapted with permission from Watarai et al., J Clin Invest, 2010 [35], Copyright (2010) American Society for Clinical Investigation

Importantly, iNKT cell development in mice was shown by using *Tcr* transgenics and iNKT cell-derived cloned mice (iNKT clone mice) to fully depend on iNKT cell-specific TCR usage (Vα14 Jα18 paired with Vβ8.2, 7, or 2) [32, 33]. Consistent with this observation, embryonic stem cells (ESCs) derived from iNKT clones, in which the *Tcr* loci were pre-rearranged in iNKT cell-specific configurations, were shown to exclusively differentiate into iNKT cell-like T cells under our in vitro differentiation protocol and exhibited similar phenotypes to conventional iNKT cells (Fig. 2) [34]. However, as ESC derivation requires embryos or donor oocytes, ESC-derived iNKT cells were expected to be unsuitable in the human clinical setting. Instead, iPSCs were considered a better source to generate iNKT cells, if iPSCs could be derived from iNKT cells. We therefore tested this possibility by using mouse iPSCs; first, those derived from mouse embryonic fibroblasts (MEFs) of iNKT clone mice and, second, those from splenic iNKT cells [35]. Despite their distinct cellular origin, they possessed the same iNKT cell-specific *Tcr* configuration and could efficiently re-differentiate into iNKT-like cells using a 25-day culture on OP9-DLL1 feeder cells supplemented with IL-7 and *Flt-3* ligand (Flt3L). Importantly, iNKT-like cells derived from iPSCs exhibited functional properties similar to splenic iNKT cells. Upon stimuli with αGalCer/DCs, they proliferated and produced cytokines, such as IFNγ, IL-4, IL-5, IL-10, and IL-13, at levels comparable to splenic iNKT cells. IPSC-derived iNKT cells were found to stably repopulate in tissues of *J*α*18*-KO, iNKT cell-deficient, mice upon adoptive transfer and to activate host NK cells after their activation by αGalCer/DCs (Fig. 3a). Moreover, in the experimental cancer therapy model, iPSC-derived iNKT cells were shown to contribute to tumor rejection by inducing adequate numbers of functional antigen-specific CD8 + cytotoxic T cells (Fig. 3b). Therefore, the mouse iPSC-derived iNKT cells were shown to retain anti-tumor adjuvant activity to a similar extent as the parental iNKT cells. These results further supported the clinical potential of the iPSC-derived iNKT cells.

## Generation of human iNKT cells from iPSCs

**Fig. 4:**
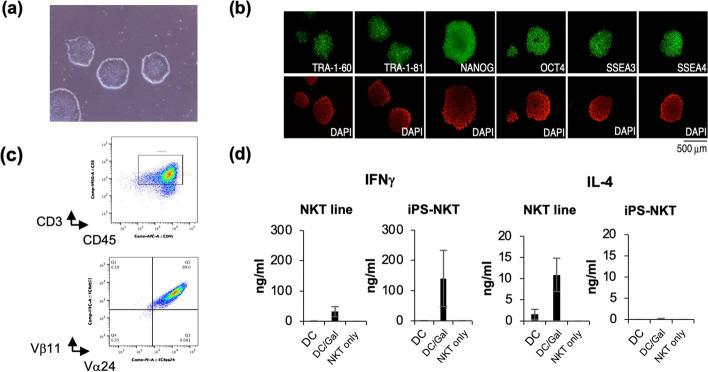
Development of human iNKT cell-derived iPSCs and their redifferentiation into iPSC-iNKT cells. **a** Colonies of human NKT-iPSCs. **b** Pluripotency of human iPSCs derived from peripheral NKT cells. **c** Flow cytometry analysis of iPSC-iNKT cells proliferated in response to hIL-7/hIL-15 stimulation. **d** Cytokine production by iPSC-iNKT cells. Figures are adapted with permission from Yamada et al., Stem Cells, 2016 [36], Copyright (2016) Oxford University Press

After the proof of concept in mice, we extended the study to investigate whether human iNKT cells could be reprogrammed to produce iPSCs and, if so, whether the iNKT cell-derived iPSCs could re-differentiate into functional iNKT cells [36]. To overcome the problem of the small number of iNKT cells in adult peripheral blood mononuclear cells (PBMCs), iNKT cells were expanded by culturing adult PBMCs in the presence of αGalCer and human IL-2 (hIL-2) and, later, cultured with αGalCer/murine DCs in the presence of hIL-2, human IL-7 (hIL-7), and human IL-15 (hIL-15) to facilitate their sustainable growth. Three days after the last re-stimulation with αGalCer/DCs, expanded iNKT cells were infected by Sendai virus vectors harboring KLF4, OCT4, SOX2, c-MYC, and SV40 Large T antigen and were cultured on MEFs with human ESC media until ESC/iPSC-like colonies appeared (Fig. 4a). Each colony was expanded and tested for TRAV10-TRAJ18 (Vα24-Jα18) and TRBV 25–1 (Vβ11) gene rearrangements, the expression of pluripotency markers (Fig. 4b), gene expression profiles, and the ability to differentiate into three germ layers in differentiation induction assays. Based on these parameters, we concluded that human iNKT cells were fully reprogrammed to produce iNKT cell-derived iPSCs (iNKT-iPSCs).iNKT-iPSCs were further tested for their ability to re-differentiate into iNKT cells by using a modified two-step protocol, first with OP9 feeder cells and, later, OP9-DLL1 cells according to our previous report [37]. After the induction culture, we found that most of the cells expressed Vα24, Vβ11, CD3, CD45, CD44, and CD69, suggesting re-differentiation of iPSCs into iNKT cells (iPSC-iNKT cells). Similar to iNKT cells isolated from PBMCs, iPSC-iNKT cells proliferated in response to hIL-7/hIL-15 stimulation (Fig. 4c) and produced similar amounts of IFNγ upon αGalCer/murine DCs stimulation to PBMC-derived iNKT cells. However, unlike PBMC-derived iNKT cells, iPSC-iNKT cells failed to produce IL-4 (Fig. 4d). Consistently, we observed downregulation of *GATA3* and reciprocal upregulation of *SOX4* in iPSC-iNKT cells. As SOX4 protein was reported to repress *GATA3* expression and suppress Th2-skewed differentiation [38], iPSC-iNKT cells are expected to possess Th1-skewed functional properties in comparison with PBMC-derived iNKT cells.

## Preclinical studies with human iPSC-iNKT cells

**Fig. 5:**
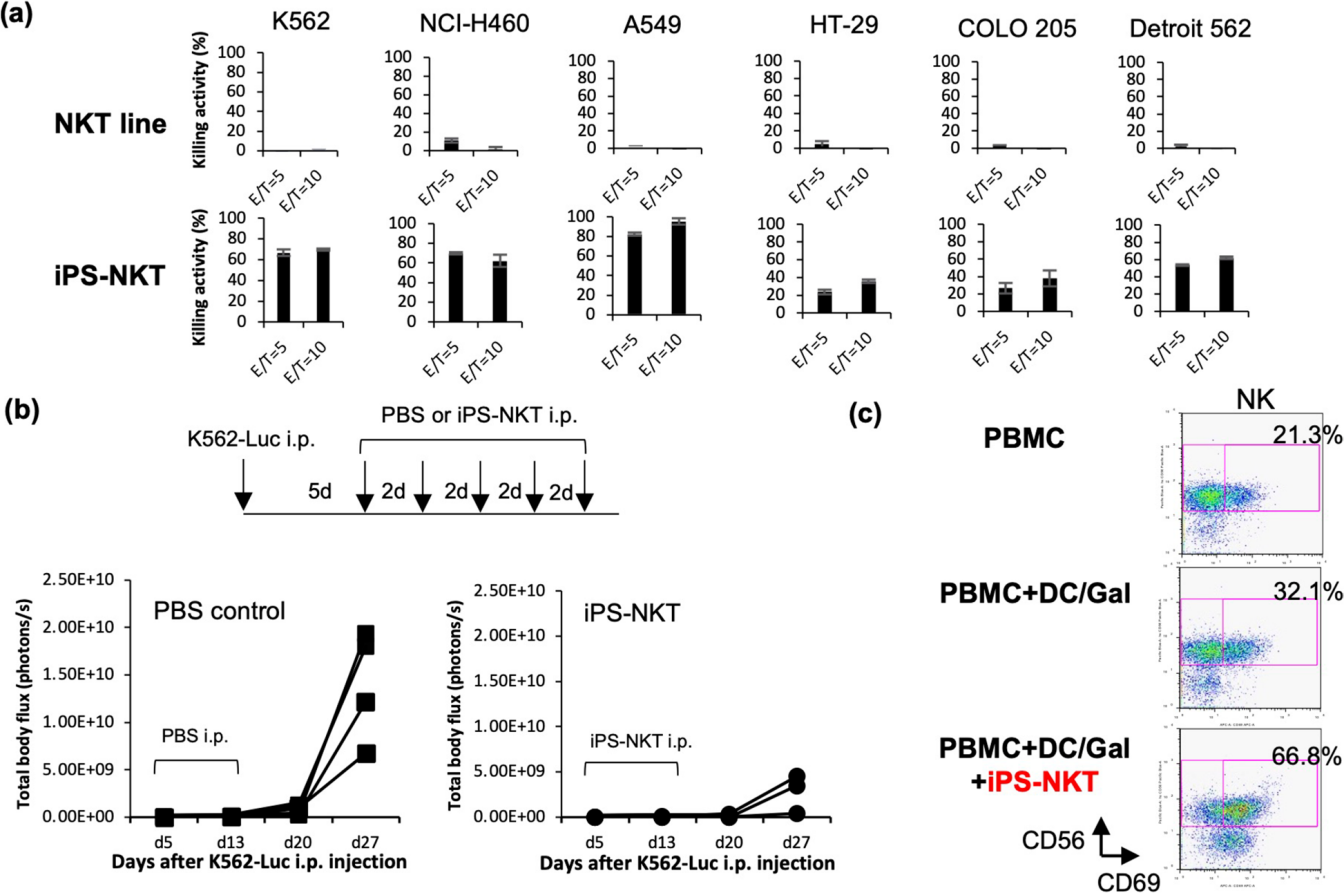
Anti-tumor activity of human iPSC-iNKT cells. **a** In vitro cytotoxicity of iPSC-iNKT cells to various cancer cell lines. **b** In vivo cytotoxicity to K562 cells in mice. **c** In vivo adjuvant activity of iPSC-iNKT cells upon stimulation of human NK cells with αGalCer/DCs. Figures are adapted with permission from Yamada et al., Stem Cells, 2016 [36], Copyright (2016) Oxford University Press

The anti-tumor potential of iPSC-iNKT cells was assessed by several parameters. The iPSC-iNKT cells were found to retain in vitro anti-tumor cytotoxic activity against six different tumor cell lines, K562, NCI-H460, A549, HT-29, COLO 205, and Detroit 562 (Fig. 5a), as was shown for primary iNKT cells. Consistently, iPSC-iNKT cells were shown to express TRAIL, Fas ligand, NKG2D, perforin, and granzyme B [36]. As EGTA/MgCl_2_ efficiently suppressed the cytotoxicity of iPSC-iNKT cells, perforin/granzyme B-mediated mechanisms could be dominantly used for their anti-tumor cytotoxicity, like primary iNKT cells [18]. This anti-tumor cytotoxic activity was further examined in tumor-bearing NOG mice, in which both acquired and innate immunity are genetically disrupted. Growth of intraperitoneally inoculated K562 cells was dampened by injection of iPSC-iNKT cells, indicating that human iPSC-iNKT cells can function as effector cells because of their intrinsic cytotoxic activity (Fig. 5b). In addition, iPSC-iNKT cells were shown to activate human NK cells upon their activation by αGalCer/DCs in NOG mice. Here, we serially transferred human PBMCs, iPSC-iNKT cells, and αGalCer/DCs into NOG mice and observed iPSC-iNKT cell-mediated activation of NK cells repopulated to the lung and liver in an αGalCer/DC-dependent manner (Fig. 5c). Moreover, activated iPSC-iNKT cells by αGalCer/DCs were revealed to be capable to induce tumor antigen-specific cytotoxic T cells in vitro [39]. We therefore conclude that human iPSC-iNKT cells can exert adjuvant activity in vivo. These preclinical studies further supported the clinical potential of human iPSC-iNKT cells for cancer immunotherapy.

## First-in-human clinical trial of iPSC-iNKT cell immunotherapy

A series of our preclinical studies provide the first proof of concept for the therapeutic potential of human iPSC-iNKT cells. This encouraged us to establish a new pipeline to produce Good Manufacturing Practice (GMP)-grade iPSC-iNKT cells, which were eventually qualified for clinical use by the Pharmaceutical and Medical Devices Agency (PMDA) in Japan. In this examination process, we were asked to pay attention to the potential tumorigenicity of the GMP-grade iPSC-iNKT cell line and also its potential risk to induce graft-versus-host disease (GVHD), infections, and infarctions due to cell aggregates. In particular, as tumorigenesis is a significant concern associated with the clinical use of iPSC-derived cell therapy, we addressed this issue in detail in the preclinical study. We indeed confirmed that iPSC-iNKT cells and even iNKT-iPSCs did not form any detectable tumors in mice, even a year after their intravenous administration. In addition, two safety criteria were used at the clinical step. First, the lack of LIN28 expression in each lot of iPSC-iNKT cells must be confirmed before shipping. Second, in the phase I trial, we selected HLA-mismatched patients for the production of iPSC-iNKT cells. Therefore, iPS-iNKT cells were expected to be rejected by the recipient patients at some point.

**Fig. 6:**
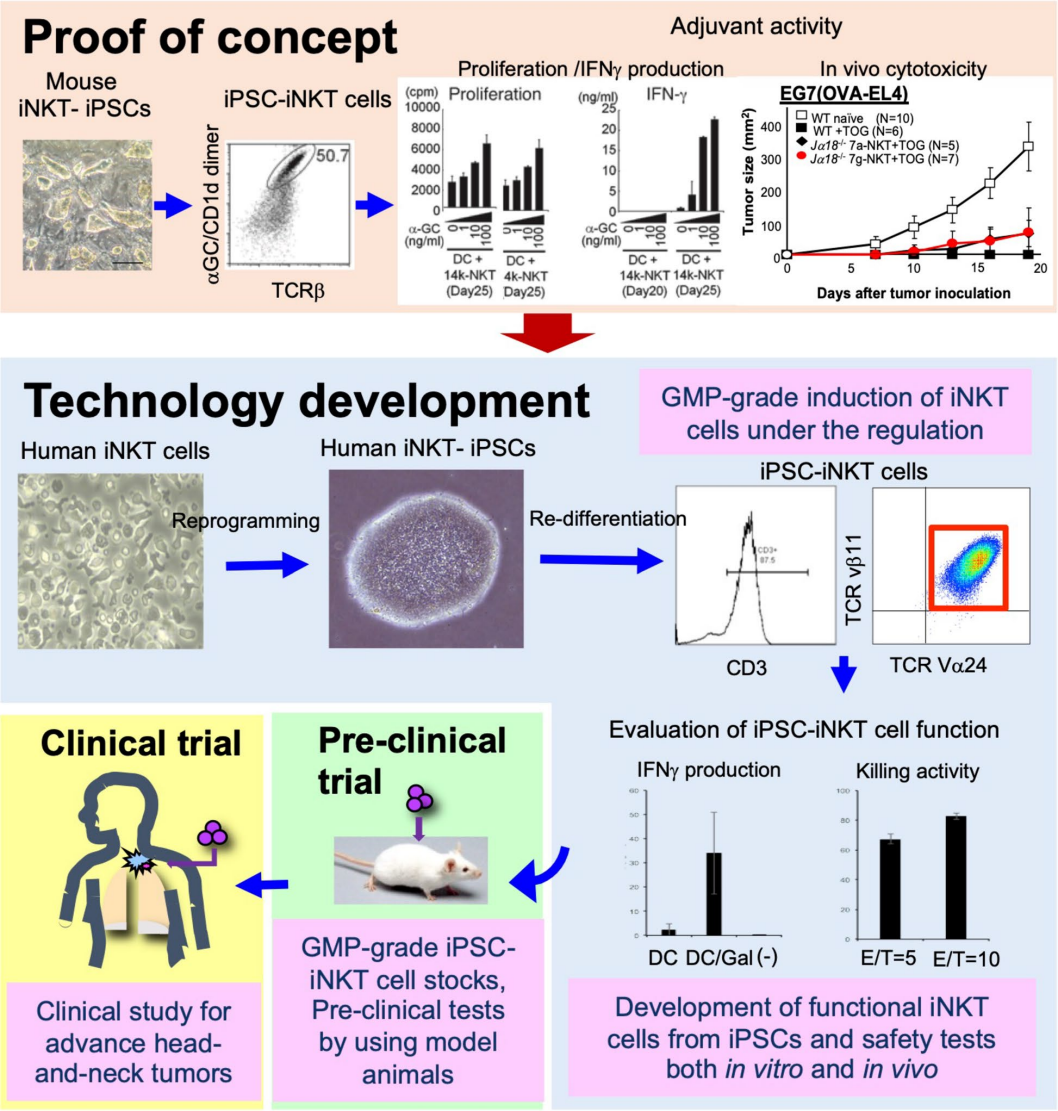
Summary of our trajectory to develop human iPSC-iNKT cells for clinical use

Upon qualification by the PMDA, we have launched a phase I clinical trial of iPSC-iNKT cell therapy for patients with squamous cell carcinoma-type advanced HNC (jRCT2033200116). Advanced HNC was chosen as a target disease because our previous clinical study, in which ex vivo expanded activated iNKT cells (5 × 10^7^) were transferred via tumor-feeding arteries in association with an intra-mucosal injection of αGalCer/APCs, supported the therapeutic potential of activated iNKT cells for HNC. We therefore set up iPSC-iNKT cell monotherapy and challenge with iPSC-iNKT cell times via tumor-feeding arteries, bi-weekly, to respective HNC patients. The primary and secondary endpoints of the study were set to prove the safety of iPSC-derived allogeneic iNKT cells and clinical efficacy, respectively. Currently, the first-in-human (FIH) clinical trial using the GMP-grade iPSC-iNKT cells (Fig. 6) is being conducted and is expected to be completed by March 2024.

## Conclusion and perspective

In this review, we have overviewed our trajectory to reach the starting line for the first clinical trial using human iPSC-iNKT cells for HNC patients. This development was initiated by our early findings demonstrating the anti-tumor activity of iNKT cells via their adjuvant activity in mice and was considerably boosted by iPSC technology in humans. Meanwhile, it is also notable that many related advances supported and were incorporated into this technology development; clinical trials to elucidate the anti-tumor activity of iNKT cells in humans, technical development to differentiate iPSCs/ESCs into iNKT cells in mice, and others. Our FIH clinical trial was initiated as a monotherapy with iPSC-iNKT cells to prove its safety. However, in this setup, the transferred iPSC-iNKT cells would not be sufficiently activated to have clinical benefit, as iNKT cells require activation by ligand-loaded APCs to exert their adjuvant activity. Therefore, it will be important to add αGalCer/APCs to the iPSC-iNKT cell therapy, once their safety in humans can be validated by the monotherapy. It would also be possible to activate the iPSC-iNKT cells by their direct recognition of tumor cells upon expression of chimeric antigen receptors (CARs) in the iPSC-iNKT cells. This, however, may need further POC studies for their adjuvant activity. Not only CARs, but also iNKT-iPSCs could provide a new platform for gene-editing technologies in the future to enhance the immunological functions of iPSC-iNKT cells in humans.

Importantly, iPSC-iNKT cells exert anti-tumor effects via distinct mechanisms from those of previously approved immunotherapies such as CAR-expressing T cell therapy and immune checkpoint inhibitors (ICIs). CAR-expressing T cells are exclusively targeted to cancers that express particular antigens. Different from them, iPSC-NKT cells exert cytotoxicity against a wide range of cancers by inducing antigen-specific cytotoxic T cells for unknown antigens and also by activating NK cells for cancers lacking the expression of HLA. ICIs are effective for a subset of patients, but the response rate is not necessarily very high. Maybe, tumor-reactive T cells are not always sufficiently generated in patients. As iPSC-iNKT cells facilitate the induction of antigen-specific cytotoxic T cells, iPSC-iNKT cell-mediated immunotherapy is expected to improve the efficacy of ICIs. Therefore, iPSC-iNKT cells could be applied in combination with ICIs and maybe other immunotherapies in the future. Indeed, iNKT cell-targeted immunotherapy model in mice revealed enhancement of the anti-tumor effect by anti-PD-1 [40].

The use of iPSC-derived immune cells for cancer immunotherapy was “a pie in the sky” a decade ago but is becoming a “realistic” treatment option. It is particularly fascinating that iPSC technology allowed us to generate abundant functional immune cells in a uniform manner as “off-the-shelf” cell products. Moreover, not only iNKT cells but also antigen-specific cytotoxic T cells and NK cells were successfully derived from human iPSCs [37, 41, 42]. These iPSC-derived T cells and NK cells were revealed to retain the anti-tumor properties of original cell types like the adjuvant activity of iPSC-NKT cells. We and other groups are performing clinical studies to warrant the safety of iPSC-derived immune cells. It is however also needed to enhance their clinical efficacy and develop more efficient and easier protocols to differentiate iPSCs into respective cells to facilitate the implementation of this technology.

## Data Availability

Not applicable.

## Abbreviations

iNKT: Invariant natural killer T
iPSC: Induced pluripotent stem cell
MHC: Major histocompatibility complex
TCR: T cell receptor
αGalCer: α-Galactosylceramide
POC: Proof of concept
iPSC-iNKT: IPCS-derived iNKT
GMP: Good manufacturing practice
αGalCer/DCs: αGalCer-pulsed dendritic cells
INFγ: Interferon-γ
NSCLC: Non-small cell lung carcinoma
αGalCer/APCs: αGalCer-pulsed antigen-presenting cells
MST: Median survival time
HNC: Head and neck cancer
PR: Partial response
SD: Stable disease
ESC: Embryonic stem cell
MEFs: Mouse embryonic fibroblasts
Flt3L: Flt-3 ligand
PBMC: Peripheral blood mononuclear cell
hIL: Human interleukin
iNKT-iPSCs: INKT cell-derived iPSCs
PMDA: Pharmaceutical and Medical Devices Agency
GVHD: Graft-versus-host disease
FIH: First-in-human
CAR: Chimeric antigen receptor
ICIs: Immune checkpoint inhibitors
OVA: Ovalbumin
